# Treatment of Inferior Vena Cava Thrombosis by Endovascular Stenting: A Case Report

**DOI:** 10.7759/cureus.19612

**Published:** 2021-11-15

**Authors:** Usman Sarwar, Nikky Bardia, Maulikumar Patel, Bassam Omar, Christopher Malozzi, Amod Amritphale, Ghulam Awan

**Affiliations:** 1 Division of Cardiology, University of South Alabama, Mobile, USA

**Keywords:** endovascular procedures, internal jugular vein, computed tomography, deep vein thrombosis, inferior vena cava

## Abstract

Optimal treatment of inferior vena cava (IVC) thrombosis remains unclear, especially given the contraindications to anticoagulation use and because interventional options remain limited. We present a case of a 62-year-old man with advanced liver cirrhosis who developed IVC thrombosis with symptoms of severe abdominal pain and leg swelling. IVC flow was restored via successful recanalization with a transjugular and common femoral approach after deploying a 22 × 70 mm Wallstent. On follow-up, the patient had a resolution of his symptoms.

## Introduction

Inferior vena cava (IVC) thrombosis can lead to considerable enfeebling. There are many causes for thrombosis forming in the venous system [1], some of the most common being inherited thrombophilia, malignancy, prolonged immobilization, and cirrhosis. Although the mainstay of acute thrombosis is anticoagulation, many patients are not candidates for anticoagulation, especially those with advanced liver cirrhosis or coexisting esophageal varices. Until recently, the treatment for acute thrombosis if anticoagulation failed was a surgical bypass of the obstructive vein, which carries an elevated risk of mortality and morbidity and a high failure rate. Now, however, with advancements in endovascular techniques, percutaneous angioplasty with stenting is the preferred treatment of choice [2,3].

## Case presentation

The patient was a 62-year-old male with a past medical history of liver cirrhosis secondary to hepatitis C, tobacco use, and post-stent coronary artery disease, who initially came to the hospital for elective left and right heart catheterization as a pre-transplant evaluation. Physical examination showed abdominal distension and diffuse tenderness with the presence of prominent superficial abdominal veins. A computed tomography (CT) scan of the abdomen with contrast was obtained immediately. The CT showed an occlusive thrombus of the IVC extending from the renal veins to the level of the cavoatrial junction. Thrombus was also observed in the portal vein, and multiple subcutaneous varicosities were found. Initially, a plan was made to start the patient on anticoagulation, but because of the patient’s history of advanced cirrhosis, large esophageal varices on recent endoscopy, and thrombocytopenia, we concluded that the patient was not a candidate for anticoagulation. Given that the patient had significant abdominal distention with pain that did not improve even after therapeutic paracentesis; we inserted a stent in the IVC to relieve the patient’s pain as a palliative procedure to improve his quality of life.

**Figure 1:**
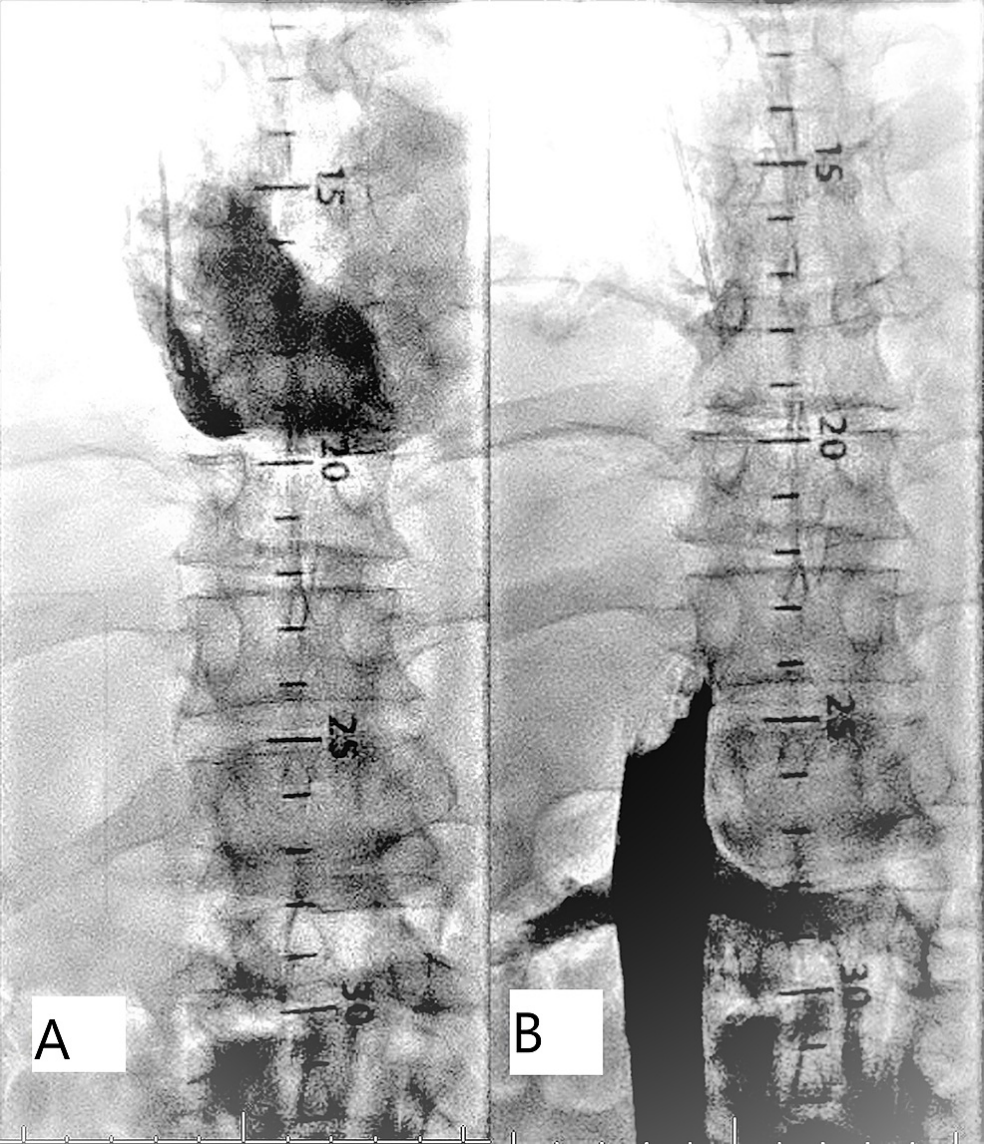
(A, B) Injection of iodine contrast showing an occluded IVC at the level of the right atrium and above the renal veins in various angiographic views IVC - inferior vena cava

Right internal jugular (IJ) and right femoral vein accesses were obtained for the procedure. A 6F pigtail diagnostic catheter was advanced from the right IJ to the right atrium, and contrast was injected into the right atrium that showed an occluded IVC at the junction of the right atrium. Another pigtail catheter was advanced through the right femoral vein and an inferior venogram was performed that showed the IVC was 100% occluded 2 cm above the renal veins (Figures 1A, 1B).

**Figure 2:**
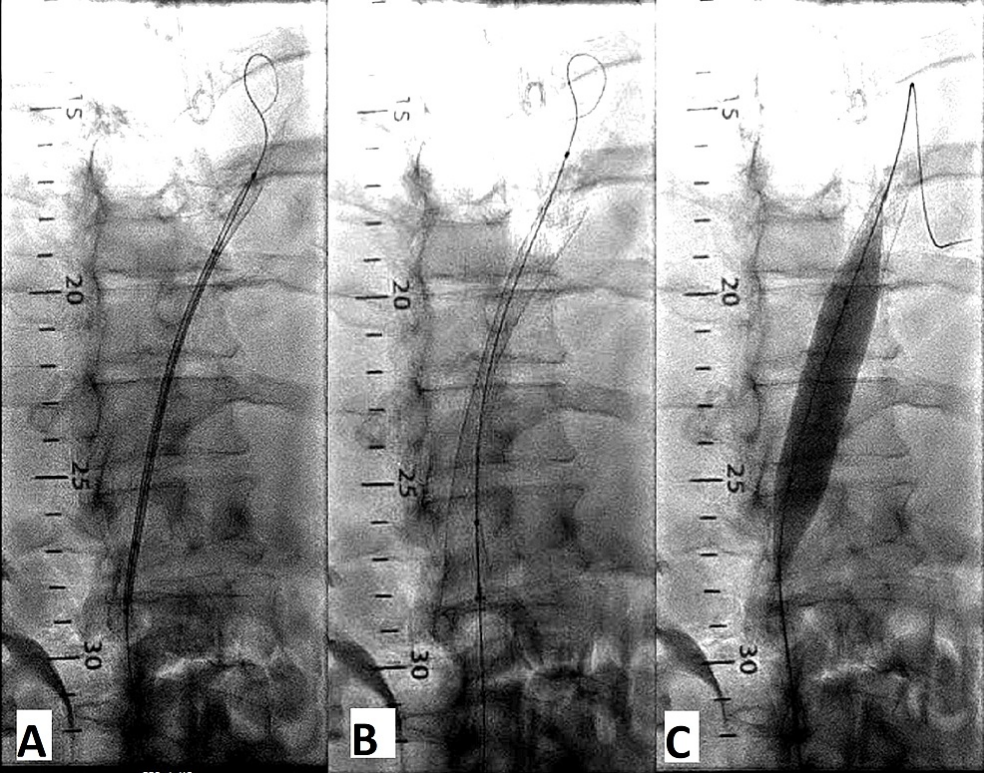
Successful wiring of IVC thrombus (A), Wallstent deployed (B), and post-dilation with Boston Scientific esophageal balloon (C)

A Glidewire advantage was advanced through the right femoral vein but was unable to cross the 100% occluded IVC. Then, a 7-French Swan-Ganz catheter was tried and was successfully advanced through the IVC all the way to the right atrium. A Swan wire was inserted through the Swan-Ganz catheter. Then, a multipurpose catheter was advanced over the Swan wire into the right atrium, and the Swan-Ganz wire was successfully replaced with a Glidewire Advantage (Figure 2A). Initially, a 9 × 80 Mustang balloon was used to perform the IVC ballooning; after IVC ballooning; however, the inferior venogram showed no flow to the IVC, which was still 100% occluded. Then, a 22 × 70 mm Wallstent was advanced via the right femoral vein to the right atrial side. The stent was deployed with a considerable waist in the mid area (Figure 2B). After stent placement, an 18 × 60 Boston Scientific esophageal balloon was successfully used to post-dilate the stent (Figure 2C).

**Figure 3:**
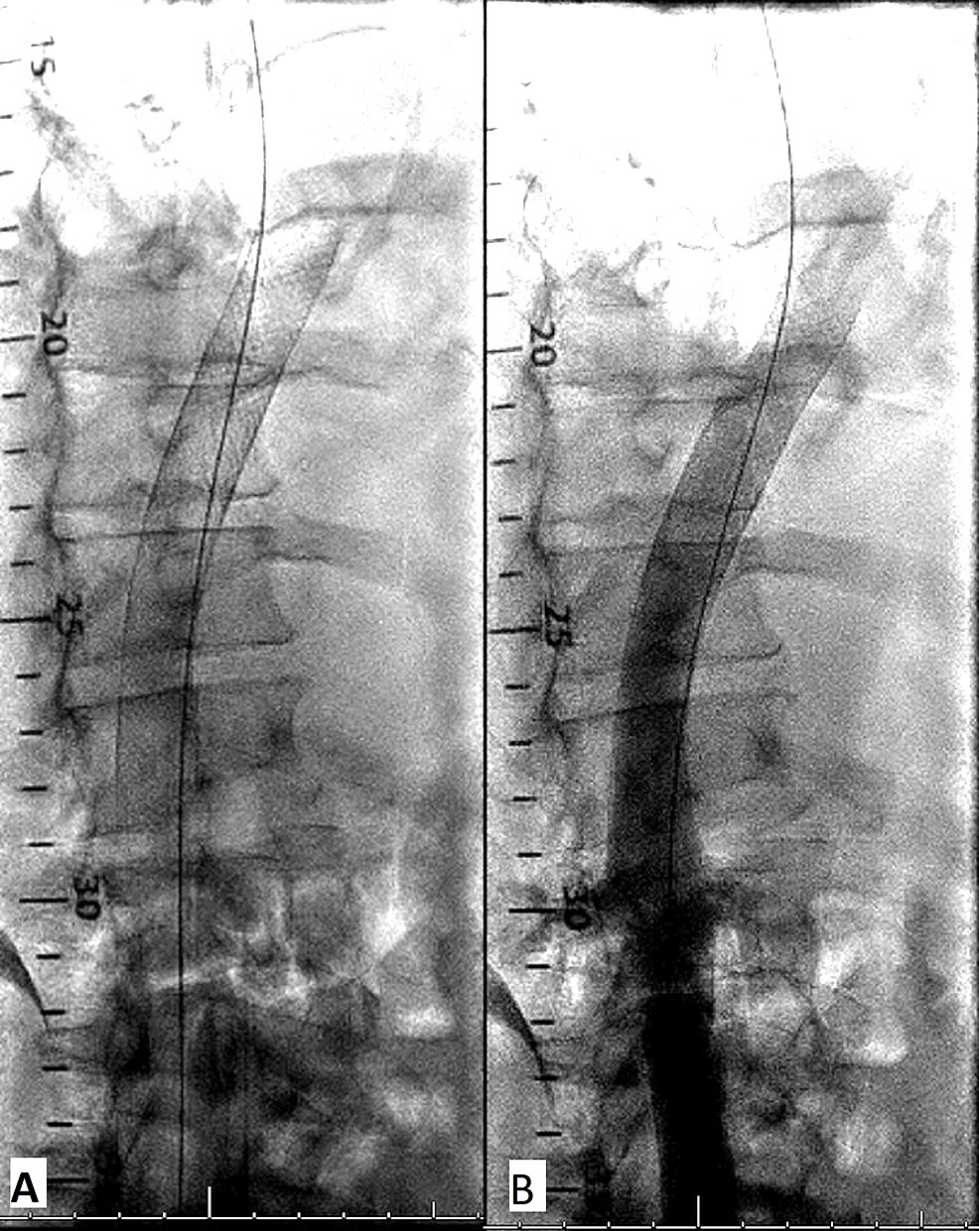
(A, B) Patent IVC after stent deployment on normal angiographic views

The final venogram (Figures 3A, 3B) and digital subtraction imaging mode showed excellent flow in the IVC to the right atrium. Small clots floating around at the lower edge of the stent above the renal artery were successfully removed with a 7-Fr snare. Usually, if IVC occlusion is of thrombosis in etiology anticoagulation is advised after venous stent placement, since our patient had an advanced liver failure with elevated INR, anticoagulation was not started [4].

## Discussion

We have described a patient with a past medical history of liver cirrhosis, now presenting with abdominal distension and significant pain due to IVC thrombus. The multistep intervention approach achieved patency of the IVC and symptomatic relief upon short-term follow-up. IVC thrombosis can cause severe complications if left untreated [5].

Initially, the mainstay of treatment had been conservative therapy with anticoagulation. If the treatment failed, however, a surgical approach with bypass was employed, which carries high morbidity and mortality. Now, due to recent advancements in percutaneous intervention techniques, venous stenting, especially in larger veins (IVC and iliac), has been proven to relieve outflow obstruction with an improvement of post-thrombotic syndrome. Studies have shown that venous stenting has a high patency rate at 12 months, with a low complication rate and good procedural success [6,7].

## Conclusions

Acquired IVC thrombosis can result from deep vein thrombosis extension into the IVC, spontaneous thrombus formation, venous stasis due to external compression, or thrombosis related to a prior IVC filter when anticoagulation is contraindicated. Interventional techniques appear to be a feasible approach with acceptable risk and symptom relief.
